# Robotic device-assisted knee extension training during the early postoperative period after opening wedge high tibial osteotomy: a case report

**DOI:** 10.1186/s13256-017-1367-3

**Published:** 2017-08-05

**Authors:** Tomokazu Yoshioka, Shigeki Kubota, Hisashi Sugaya, Kojiro Hyodo, Kaishi Ogawa, Yu Taniguchi, Akihiro Kanamori, Yoshiyuki Sankai, Masashi Yamazaki

**Affiliations:** 1 Division of Regenerative Medicine for Musculoskeletal System, Faculty of Medicine, University of Tsukuba, 1-1-1 Tennodai, Tsukuba, Ibaraki 305-8575 Japan; 2 Department of Orthopedic Surgery, Faculty of Medicine, University of Tsukuba, 1-1-1 Tennodai, Tsukuba, Ibaraki 305-8575 Japan; 3 Faculty of Systems and Information Engineering, University of Tsukuba, 1-1-1 Tennodai, Tsukuba, Ibaraki 305-8577 Japan

**Keywords:** Opening wedge high tibial osteotomy, Rehabilitation, Range of motion, Wearable robot suit

## Abstract

**Background:**

Maintenance or restoration of a good range of motion of the knee is one of the most important outcomes following knee surgery. According to previous studies, opening wedge high tibial osteotomy enables better recovery of range of motion in knee flexion than that achievable after total knee arthroplasty or unicompartmental knee arthroplasty. However, few reports provide a detailed description of the postoperative recovery of knee extension range of motion after opening wedge high tibial osteotomy. We describe our experience with a knee extension training program using a single-joint hybrid assistive limb device (HAL-SJ; Cyberdyne Inc., Tsukuba, Japan) during the acute recovery phase after opening wedge high tibial osteotomy. The HAL-SJ is a wearable robotic device that facilitates voluntary control of knee joint motion.

**Case presentation:**

A 67-year-old Japanese woman who underwent opening wedge high tibial osteotomy for spontaneous osteonecrosis of the left medial femoral condyle received HAL-SJ-based knee extension training postoperatively. Our experience with this patient revealed that knee extension training with the HAL-SJ during the acute phase following opening wedge high tibial osteotomy is feasible. Furthermore, the patient’s knee extension range of motion improved to values similar to those seen during the preoperative stage, and her flexion range of motion was improved at 3 months after the surgery.

**Conclusions:**

HAL-SJ-based knee extension training could be used as a novel post-opening wedge high tibial osteotomy rehabilitation modality. Further exploration of individualized optimal settings of the HAL-SJ is required to improve its safety and efficacy.

## Background

Opening wedge high tibial osteotomy (OWHTO) is a surgical procedure whereby tibial osteotomy is performed in the proximal tibial tuberosity from the medial to the lateral side. OWHTO is performed to treat osteoarthritis of the knee and spontaneous osteonecrosis of the knee (SPONK) localized to the medial compartment, in order to correct varus deformity of the knee joint and lower limb alignment [1]. Currently, in Japan, OWHTO is a commonly performed joint-sparing surgery that preserves physiological knee joint function, which is particularly important in Japanese patients because the Japanese lifestyle includes activities of daily living performed while sitting on the floor, with the knee in full flexion and the buttocks resting on the heels. Further advantages of OWHTO include easy inclusion of an internal fixation device, enhancement of postoperative treatments, and better recovery or maintenance of knee range of motion (ROM) than that achievable after total knee arthroplasty (TKA) [2]. However, because osteotomy of the proximal tibial tuberosity results in its enlargement, the patella typically descends after OWHTO [3, 4], which increases the patellofemoral joint pressure [5], increases anterior knee pain [6], and influences the knee extensor mechanism. Staubli *et al*. [1] reported that the range of passive motion of the knee reached baseline values at the 12-week follow-up examination. Although OWHTO can enable better recovery of knee flexion ROM, it is difficult to improve knee extension ROM with OWHTO. Therefore, the influence of OWHTO on the knee extensor mechanism remains of concern, and it is necessary to minimize such effects during rehabilitation. For this purpose, it is desirable to develop new rehabilitation intervention strategies that can be implemented during the early postoperative period without increasing anterior knee pain. Our previous case report described knee joint extension training using the single-joint hybrid assistive limb device (HAL-SJ; Cyberdyne Inc., Tsukuba, Japan) to provide rehabilitation treatment targeted to improving knee joint function after TKA [7]. In this previously described case, we obtained immediate improvement of the extension lag (EL) without increasing knee pain [7]. The HAL-SJ is a wearable robotic device that facilitates the voluntary control of knee joint motion. With this device, signals from muscle action potentials are detected through electrodes on the surface of the skin and processed through a computer, which then assists the patient in performing the intended joint motions. The power unit on the knee joint comprises angular sensors and actuators, and the control system comprises a cybernetic voluntary control (CVC) system and a cybernetic autonomous control system [8]. The HAL-SJ has been reported to be effective in the functional recovery of various mobility disorders [9–12]. Although study researchers have reported successful outcomes for acute or chronic mobility disorders, to date, there have been no reports of the use of HAL-SJ for postoperative recovery following OWHTO. Accordingly, we report the experience of one of our patients with a HAL-SJ-based knee extension training program during the acute recovery phase after OWHTO.

## Case presentation

**Fig. 1:**
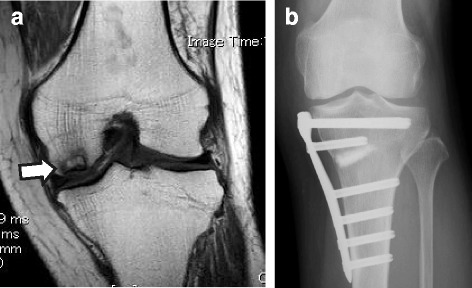
Frontal images of the knee joint in a 67-year-old woman who underwent opening wedge high tibial osteotomy for osteonecrosis of the knee. **a** The lesion area visualized by preoperative magnetic resonance imaging is shown with an *arrow*. **b** Postoperative radiograph

A 67-year-old Japanese woman underwent OWHTO to treat SPONK that had occurred in the left medial femoral condyle (Fig. 1). The patient reported the following history of the present illness. Pain in the left knee joint initially occurred at 1 year and 4 months before admission, without any particular trigger. Her former treating physician had provided conservative treatments such as oral administration of anti-inflammatory analgesics, rehabilitation including quadriceps muscle training, and guidance on the use of a cane to relieve weight bearing. However, this treatment was not successful in relieving her symptoms, and she was referred to our department for surgery. The patient had a history of hypertension and hyperlipidemia, and she had been taking oral candesartan and atorvastatin. The patient was a housewife with no noteworthy aspects related to social, family, or environmental history. On physical examination, the patient had a height of 157 cm, a body weight of 62 kg, and a body mass index of 25.2 kg/m^2^. She reported feeling pain in the medial joint space of the knee during walking, as well as during climbing or descending stairs. At the time of the first admission, her systolic/diastolic blood pressure was 165/97 mmHg. Table 1 provides an overview of the patient’s pain scores in the knee joint, ROM of the knee joint, and knee joint function. Neurological examination revealed no abnormal findings. Similarly, laboratory findings at the time of the first admission did not indicate any abnormality: total protein 7.3 g/dl, albumin 4.6 g/dl, aspartate aminotransferase 22 U/L, alanine aminotransferase 18 U/L, lactate dehydrogenase 213 U/L, alkaline phosphatase 297 U/L, γ-glutamyl transferase 18 U/L, total bilirubin 0.6 mg/dl, Na 142 mEq/L, Cl 104 mEq/L, K 4.5 mEq/L, blood urea nitrogen 21.4 mg/dl, creatinine 0.56 mg/dl, Ca 9.7 mg/dl, inorganic phosphorus 3.7 mg/dl, leukocytes 5100/μl, erythrocytes 5.00 × 10^6^/μl, hemoglobin 14.4 g/dl, hematocrit 43.6%, and platelets 263 × 10^3^/μl.

**Table 1:** Chronological changes in scores during recovery after opening wedge high tibial osteotomy

		Preoperative	First HAL-SJ (POD 8)		Second HAL-SJ (POD 10)		Third HAL-SJ (POD 15)		Fourth HAL-SJ (POD 17)		Fifth HAL-SJ (POD 22)		Sixth HAL-SJ (POD 24)		At discharge (POD 30)	After end of sixth HAL-SJ	
			IBI	IFI	IBI	IFI	IBI	IFI	IBI	IFI	IBI	IFI	IBI	IFI		1 month	3 months
EL, degrees		3	6	2	3	6	10	6	5	6	3	8	4	0	4	3	3
VAS, mm		60	49	83	29	23	12	13	12	9	6	21	8	8	6	24	69
IKEMS, kgf		17.6	5.3	5.7	6.6	6.5	7.5	8.6	9.4	9.4	11.0	12.2	13.4	13.2	12.8	18.7	16.6
Active ROM, degrees		10–135	16–124	12–125	16–124	9–120	19–133	13–136	16–138	15–134	10–139	12–139	12–139	11–136	12–144	11–135	12–142
JOA score	Total	60	N/A	N/A	N/A	N/A	N/A	N/A	N/A	N/A	N/A	N/A	N/A	N/A	55	65	85
	Pain on walking	20	N/A	N/A	N/A	N/A	N/A	N/A	N/A	N/A	N/A	N/A	N/A	N/A	15	15	25
	Pain on ascending or descending stairs	5	N/A	N/A	N/A	N/A	N/A	N/A	N/A	N/A	N/A	N/A	N/A	N/A	5	15	20
	ROM	25	N/A	N/A	N/A	N/A	N/A	N/A	N/A	N/A	N/A	N/A	N/A	N/A	25	25	30
	Joint effusion	10	N/A	N/A	N/A	N/A	N/A	N/A	N/A	N/A	N/A	N/A	N/A	N/A	10	10	10

*Abbreviations: EL* Extension lag, *VAS* Visual analogue scale, *IKEMS* Isometric knee extension muscle strength, *ROM* Range of motion, *JOA* Japanese Orthopaedic Association, *POD* Postoperative day, *HAL-SJ* Single-joint hybrid assistive limb device, *IBI* Immediately before intervention, *IFI* Immediately after intervention, *N/A* Not applicable

The HAL-SJ treatment program was divided into five phases [7]. Phase 1 consisted of preoperative observation from the day of hospital admission until the day of surgery. The patient’s thigh circumference and lower leg length were measured preoperatively to adjust the HAL-SJ to the patient’s physical size, which would ensure appropriate training. We palpated the patient’s quadriceps muscles (vastus medialis, rectus femoris, and vastus lateralis) and attached electrodes to each muscle to detect the bioelectric potentials of the long axis along the belly of each muscle. We instructed the patient to perform knee extension and to contract her quadriceps. On the basis of these data, we instructed the patient to simulate the knee extension training, which would be performed postoperatively. Specifically, the patient sat with her lower leg hanging down naturally, and the height of the chair was adjusted so that the patient’s feet were not in contact with the floor. The patient performed ten knee extensions with HAL-SJ assistance, using the muscle that exhibited the largest bioelectric potential amplitude.

**Fig. 2:**
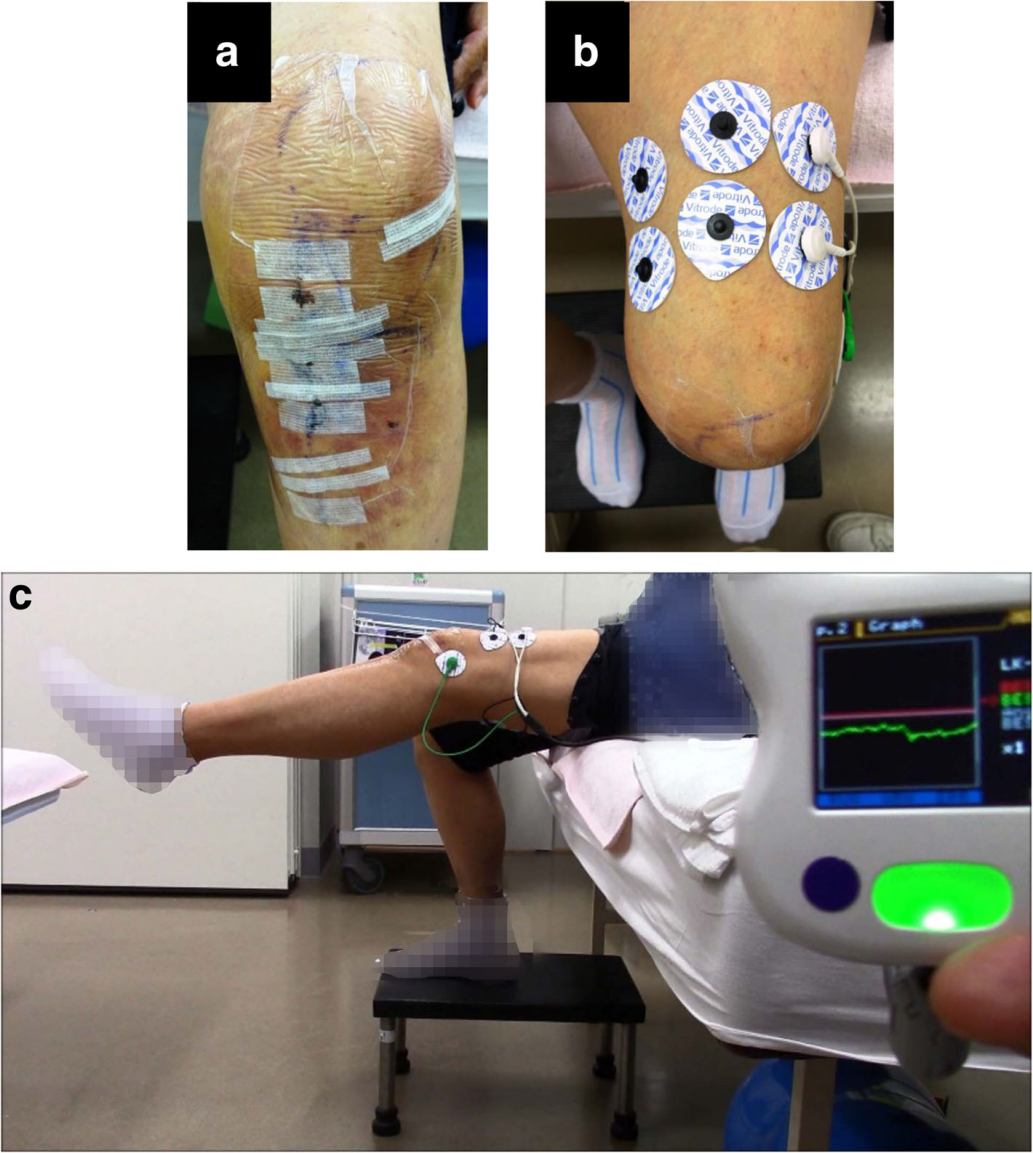
Detection of the bioelectric potential and training simulation prior to beginning single-joint hybrid assistive limb (HAL-SJ) training. **a** Overview of the operative wound. **b** One day prior to beginning the knee extension training using HAL-SJ, we attached electrodes to the quadriceps muscle. **c** We simulated knee extension training using the vastus lateralis, which exhibited the largest bioelectric potential amplitude

Phase 2 involved surgery (day of surgery). The patient underwent OWHTO (Figs. 1b and 2a) using TomoFix (DePuy Synthes, Bettlach, Switzerland), artificial bone (OSferion60, β-tricalcium phosphate; Olympus Terumo Biomaterials, Tokyo, Japan), and biplanar osteotomy, as described by Takeuchi *et al*. [2]. The preoperative weight-bearing line (WBL) percentage [2], calculated on the basis of an anteroposterior weight-bearing radiograph of the affected leg in full knee extension, was 29.6%. The surgery was planned with a target postoperative WBL percentage of 62.5%. The actual enlarged angle and distance of osteotomy were 5.5 degrees and 7 mm, respectively.

Phase 3 consisted of postoperative observation from day 1 to day 7 postoperatively. For postoperative rehabilitation, partial weight bearing was allowed after a non-weight-bearing period of 2 weeks, and full weight bearing began at 4 weeks postoperatively. The patient received rehabilitation training under the guidance of a physical therapist (ROM exercises and muscle-strengthening training) for 20–40 minutes each day, 5 days per week, from the first postoperative day until discharge. For ROM exercises, continuous passive motion training began on the first postoperative day for 1 hour per day and continued every day until discharge. On postoperative day 7, we attached electrodes to the quadriceps muscle to detect the bioelectric potential along the long axis of the rectus femoris muscle belly (Fig. 2b). Then, the patient was instructed to perform active knee extension exercises that involved contracting her quadriceps, thus simulating training with HAL-SJ (Fig. 2c).

**Fig. 3:**
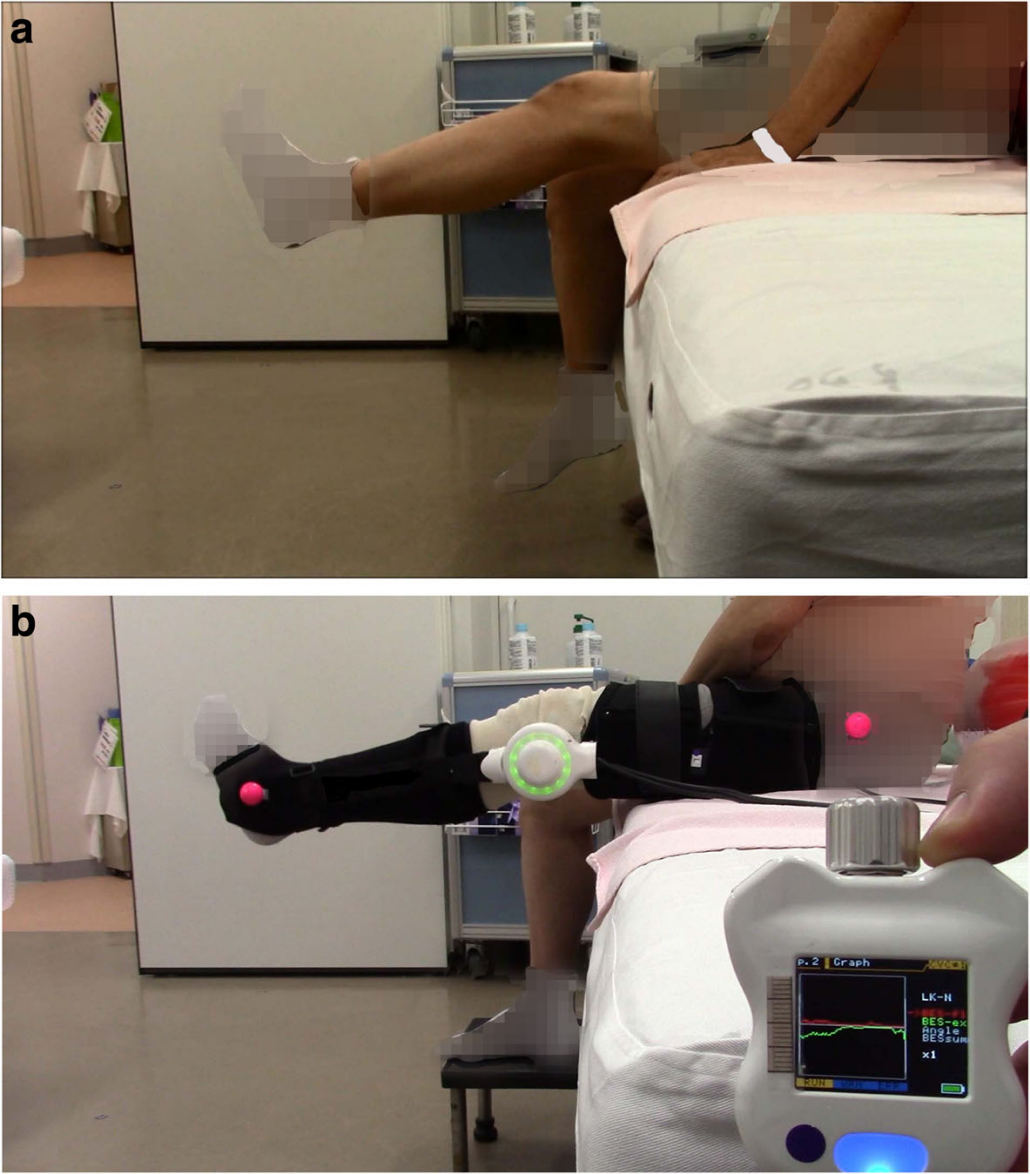
Knee extension training using the single-joint hybrid assistive limb (HAL-SJ). **a** Active knee extension. **b** Assisted knee extension training while wearing the HAL-SJ

Phase 4 consisted of HAL-SJ therapy from postoperative day 8 to discharge. The CVC mode of HAL-SJ serves to support the patient’s voluntary motion on the basis of voluntary muscle activity and the assistive torque provided to the knee joint [10]. In this study, we used the CVC mode, which allowed the operator to adjust the degree of physical support to maintain the patient’s comfort while gradually reducing support as training progressed. In addition to the regular rehabilitation program (Fig. 3a), the patient performed HAL-JS-assisted knee extension exercises while sitting, which consisted of five sets of ten repetitions per set, twice per week (Fig. 3b). The training was performed six times before the patient was discharged (postoperative days 8, 10, 15, 17, 22, and 24). The mean training duration, including the time required to put on the HAL-SJ, was 15.5 ± 1.4 minutes (15 minutes, 16 minutes, 17 minutes, 16 minutes, 13 minutes, and 16 minutes during training sessions 1, 2, 3, 4, 5, and 6, respectively). There were no adverse events related to the use of HAL-SJ, and the patient was discharged 30 days after the surgery.

**Fig. 4:**
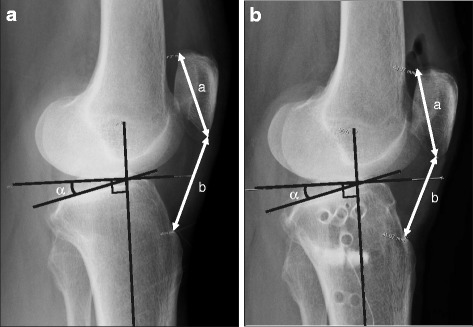
Tibial slope angle and Insall-Salvati ratio. These indicators were measured on the lateral radiograph of the knee joint. **a** Before surgery. **b** After nail extraction at 1 year after opening wedge high tibial osteotomy. *α* Tibial slope angle, *b/a* Insall-Salvati ratio

Phase 5 consisted of post-HAL-SJ therapy observation from discharge until 3 months after the end of HAL-SJ therapy, during which the following indicators were assessed: EL, assessed as the difference between the maximum knee joint extension angle during passive exercise and that during active exercise; knee pain rated on the visual analogue scale (VAS); isometric knee extension muscle strength (IKEMS); active ROM before surgery, before and after HAL-SJ training, and at 1 and 3 months after the end of training; and the Japanese Orthopaedic Association (JOA) score [13] before surgery, at discharge, and at 1 and 3 months after the end of training. Furthermore, we used lateral radiographs to measure the tibial slope angle (TSA) and Insall-Salvati ratio (ISR) as indicators of patellar tendon shortening before surgery and after implant removal at 1 year after the operation [2] (Fig. 4a, b). Measurement of knee ROM was performed using goniometry, and the landmarks used in the measurements were the greater trochanter of the femur, proximal head of the fibula, and lateral malleolus. Goniometry was used because it has been reported that goniometric measurements of ROM are more reliable than visual observation, with an accuracy of up to 1.0 degree [14]. The maximal IKEMS of the operated leg was assessed with the patient in a sitting position, with the hips and knees flexed at 90 degrees. A μTas F-1 handheld dynamometer (Anima Corp., Tokyo, Japan) was fixed to the chair, and two measurements were recorded. Each trial lasted 3–5 seconds, with 30 seconds of rest between trials. The higher score of two valid trials was recorded. All measurements were performed by a single trained physical therapist to eliminate interobserver variability. The EL, VAS, IKEMS, and ROM results are shown in Table 1. The EL improved from 3 degrees preoperatively to 0 degrees at the end of the sixth HAL-SJ training session, but it returned to 3 degrees at 1 and 3 months after the end of the training. When we compared results obtained before training against those obtained after each of the six training sessions, we observed that three sessions produced improved EL and three sessions produced improved or relatively constant VAS scores immediately after training. However, for the other three sessions, the patient reported increased pain immediately after training, with the greatest increase in pain reported immediately after the first HAL-SJ training session, when the VAS score was 1.7 times higher immediately after training than before training. The EL improved after training sessions 1 and 3, when the patient reported increased knee pain (expressed as the VAS score). However, the EL did not change after training sessions 2 and 4, when the patient reported less knee pain (improved VAS score immediately after training). The patient did not refuse to undergo training because of increased pain. The IKEMS value was the largest preoperatively (17.6 kgf) and decreased to its lowest value (30% of the preoperative strength) before the first HAL-SJ intervention at 8 days after the operation. The IKEMS remained relatively constant throughout HAL-SJ training but recovered to the preoperative value during follow-up (18.7 and 16.6 kgf at 1 and 3 months, respectively, after completion of HAL-SJ training). The active ROM also recovered to the preoperative value by the end of the sixth HAL-SJ training session. Thereafter, the extension ROM did not change, but the flexion ROM was maintained or improved. The JOA score decreased from 60 points preoperatively to 55 points at discharge, but it improved with time, reaching 65 and 85 points at 1 and 3 months, respectively, after the end of HAL-SJ training. Whereas the TSA was maintained at 13.1 degrees before and after the surgery, the ISR decreased from 1.13 preoperatively to 0.97 postoperatively. At 6 months after surgery, the passive ROM was 0–135 degrees, and the JOA score was 90 points. There were no complications throughout the preparation, surgery, or rehabilitation process.

## Discussion

The case described in the present report illustrates two important clinical suggestions. First, knee extension training using the HAL-SJ during the acute phase after OWHTO is clinically feasible. Second, although we did not find an improvement in the EL or a consistent tendency toward improvement in knee pain, extension ROM recovered to near-preoperative values, and flexion ROM improved at 3 months after the surgery.

We previously reported the case of a patient who underwent three sessions of knee extension training using the HAL-SJ as part of the inpatient acute rehabilitation program after TKA, and we also found in that case that HAL-SJ-assisted training was safe and feasible [7]. In the present application of HAL-SJ training following OWHTO, the patient’s knee pain increased after three of the six sessions (sessions 1, 3, and 5), but the intervention was not stopped because of the increase in pain, and the patient completed all six training sessions. OWHTO is a joint-sparing surgery during which lower limb alignment is osteogenically adjusted using the extracapsular approach. Unlike TKA, OWHTO does not involve direct surgical intervention into the intra-articular tissues or knee extension mechanism. However, our patient experienced increased knee joint pain, likely as a result of the increased patellofemoral joint pressure [5]. Previous studies have shown that the patella descends after OWHTO [3, 4], thus increasing patellofemoral joint pressure [5] and causing anterior knee pain [6]. Consistent with these previous reports, the ISR of our patient decreased from 1.13 preoperatively to 0.97 postoperatively, and the position of the patella was lower after than before the surgery. Furthermore, an increased TSA due to OWHTO can cause limited knee extension ROM and knee joint pain [15], although a postoperative increase in the TSA was not observed in our patient. It is also possible that pain receptors were stimulated because OWHTO is a joint-sparing surgery that preserves biological tissue. With TKA, the patient would not feel pain on the articular surface, because this surface is replaced by an artificial component.

In our patient, there was no consistent improvement in EL or decrease in knee joint pain after knee extension training using the HAL-SJ, although knee extension ROM recovered to a level similar to that before surgery, and knee flexion ROM increased 3 months after the surgery. Takeuchi *et al*. [2] described the indication for OWHTO, including having an ROM of the knee of at least 15 degrees of extension to 120 degrees of flexion. In our patient, the ROM was 10135 degrees preoperatively and 12–142 degrees at the end of the postoperative observation period. It has been shown that, compared with TKA or unicompartmental knee arthroplasty, OWHTO can result in better knee flexion ROM following a regular rehabilitation program [16], suggesting that the increased knee flexion angle noted in our patient was not related to HAL-SJ training. The immediate effect of HAL-SJ training resulting in improved EL without increasing knee joint pain, which is typically observed after TKA [7], was not consistently found in our patient who underwent OWHTO. Instead, we found a different pattern: During the first four of six sessions, the EL improved when knee joint pain increased immediately after the training, whereas the EL did not improve when knee pain did not increase. Further investigation with a larger sample size is warranted to determine the optimal settings for the HAL-SJ assist function to improve the EL without increasing knee joint pain. Authors of a recent study comparing the outcomes of a HAL-SJ rehabilitation program with those of regular rehabilitation during the acute phase after TKA [17] reported that the HAL-SJ group experienced greater improvement in EL and a lower incidence of knee joint pain during training.

The basic differences between TKA and OWHTO are related to the fact that TKA is a surgical procedure in which the internal structures of the joint are entirely replaced with an artificial joint, whereas OWHTO is a surgical procedure in which only the alignment of the load axis is corrected, and the correction is performed extraarticularly, while the degenerated joint’s internal structure is preserved. Even in patients with limited knee extension preoperatively, the amount of bone cut during TKA and the technique used for the detachment of soft tissues allow for improvement in knee extension. Meanwhile, because OWHTO involves alignment correction through extraarticular osteotomy, any limitation of joint extension already present before surgery is difficult to improve through OWHTO. During HAL-SJ training, the assistance provided by the robotic device helps improve the range of active extension to approximately the range of passive extension, minimizing EL. In other words, because OWHTO is less likely to bring an improvement in the range of passive extension, it may be difficult to demonstrate the beneficial effect of post-OWHTO training using the HAL-SJ. In Japan, improvement in knee flexion ROM is of particular importance because certain aspects of the Japanese lifestyle involve activities such as sitting on the floor with the knees in full flexion and the buttocks resting on the heels. Detailed investigations regarding this topic have been performed [2, 16], but no studies have reported the preoperative to postoperative recovery of knee extension ROM. The present case report is, to the best of our knowledge, the first to describe the application of the HAL-SJ wearable robotic device for knee extension training during the acute phase after OWHTO and to be focused on the recovery of knee extension ROM.

## Conclusions

The observations in our patient indicated that knee extension training using the HAL-SJ is feasible during the acute phase after OWHTO. Unlike TKA, which involves replacement of the degenerated and deformed intraarticular structure with a compatible artificial component, OWHTO is a surgical procedure whose aim is to adjust lower limb alignment while preserving the joint via osteotomy. Therefore, complete knee extension cannot be obtained through OWHTO. Further exploration regarding individualized optimal settings of the HAL-SJ is required to improve the safety and efficacy of HAL-SJ training in the acute phase after knee joint surgery.

## Abbreviations

CVC: Cybernetic voluntary control
EL: Extension lag
HAL-SJ: Single-joint hybrid assistive limb device
IBI: Immediately before intervention
IFI: Immediately after intervention
IKEMS: Isometric knee extension muscle strength
ISR: Insall-Salvati ratio
JOA: Japanese Orthopaedic Association
N/A: Not applicable
OWHTO: Opening wedge high tibial osteotomy
POD: Postoperative day
ROM: Range of motion
SPONK: Spontaneous osteonecrosis of the knee
TKA: Total knee arthroplasty
TSA: Tibial slope angle
VAS: Visual analogue scale
WBL: Weight-bearing line

## References

[1] Staubli AE, De Simoni C, Babst R, Lobenhoffer P. TomoFix: a new LCP-concept for open wedge osteotomy of the medial proximal tibia – early results in 92 cases. Injury. 2003;34 Suppl 2:B55–62. 10.1016/j.injury.2003.09.025 14580986

[2] Takeuchi R, Ishikawa H, Aratake M, Bito H, Saito I, Kumagai K, *et al.* Medial opening wedge high tibial osteotomy with early full weight bearing. Arthroscopy. 2009;25:46–53. 10.1016/j.arthro.2008.08.015 19111218

[3] Gaasbeek R, Welsing R, Barink M, Verdonschot N, van Kampen A. The influence of open and closed high tibial osteotomy on dynamic patellar tracking: a biomechanical study. Knee Surg Sports Traumatol Arthrosc. 2007;15:978–84. 10.1007/s00167-007-0305-0 17483931

[4] Bito H, Takeuchi R, Kumagai K, Aratake M, Saito I, Hayashi R, *et al.* Opening wedge high tibial osteotomy affects both the lateral patellar tilt and patellar height. Knee Surg Sports Traumatol Arthrosc. 2010;18:955–60. 10.1007/s00167-010-1077-5 20217394

[5] Stoffel K, Willers C, Korshid O, Kuster M. Patellofemoral contact pressure following high tibial osteotomy: a cadaveric study. Knee Surg Sports Traumatol Arthrosc. 2007;15:1094–100. 10.1007/s00167-007-0297-9 17342550

[6] Song IH, Song EK, Seo HY, Lee KB, Yim JH, Seon JK. Patellofemoral alignment and anterior knee pain after closing- and opening-wedge valgus high tibial osteotomy. Arthroscopy. 2012;28:1087–93. 10.1016/j.arthro.2012.02.002 22520445

[7] Yoshioka T, Sugaya H, Kubota S, Onishi M, Kanamori A, Sankai Y, *et al.* Knee-extension training with a single-joint hybrid assistive limb during the early postoperative period after total knee arthroplasty in a patient with osteoarthritis. Case Rep Orthop. 2016;2016:9610745. 10.1155/2016/9610745 PMC5059619 27774330

[8] Kawamoto H, Sankai Y. Power assist method based on phase sequence and muscle force condition for HAL. Adv Robot. 2005;19:717–34.

[9] Kawamoto H, Kamibayashi K, Nakata Y, Yamawaki K, Ariyasu R, Sankai Y, *et al.* Pilot study of locomotion improvement using hybrid assistive limb in chronic stroke patients. BMC Neurol. 2013;13:141. 10.1186/1471-2377-13-141 PMC3851710 24099524

[10] Kubota S, Nakata Y, Eguchi K, Kawamoto H, Kamibayashi K, Sakane M, *et al.* Feasibility of rehabilitation training with a newly developed wearable robot for patients with limited mobility. Arch Phys Med Rehabil. 2013;94:1080–7. 10.1016/j.apmr.2012.12.020 23313372

[11] Fujii K, Abe T, Kubota S, Marushima A, Kawamoto H, Ueno T, *et al.* The voluntary driven exoskeleton Hybrid Assistive Limb (HAL) for postoperative training of thoracic ossification of the posterior longitudinal ligament: a case report. J Spinal Cord Med. 2017;40:361–7. 10.1080/10790268.2016.1142056 PMC5472024 26856189

[12] Ikumi A, Kubota S, Shimizu Y, Kadone H, Marushima A, Ueno T, *et al.* Decrease of spasticity after hybrid assistive limb® training for a patient with C4 quadriplegia due to chronic SCI. J Spinal Cord Med. doi:10.1080/10790268.2016.1225913. PMC5815155 27762171

[13] Okuda M, Omokawa S, Okahashi K, Akahane M, Tanaka Y. Validity and reliability of the Japanese Orthopaedic Association score for osteoarthritic knees. J Orthop Sci. 2012;17:750–6. 10.1007/s00776-012-0274-0 22868702

[14] Lenssen AF, van Dam EM, Crijns YH, Verhey M, Geesink RJ, van den Brandt PA, *et al.* Reproducibility of goniometric measurement of the knee in the in-hospital phase following total knee arthroplasty. BMC Musculoskelet Disord. 2007;8:83. 10.1186/1471-2474-8-83 PMC2040146 17705860

[15] Pipino G, Indelli PF, Tigani D, Maffei G, Vaccarisi D. Opening-wedge high tibial osteotomy: a seven - to twelve-year study. Joints. 2016;4:6–11. 10.11138/jts/2016.4.1.006 PMC4914376 27386441

[16] Takeuchi R, Umemoto Y, Aratake M, Bito H, Saito I, Kumagai K, *et al.* A mid term comparison of open wedge high tibial osteotomy vs unicompartmental knee arthroplasty for medial compartment osteoarthritis of the knee. J Orthop Surg Res. 2010;5:65. 10.1186/1749-799X-5-65 PMC2940897 20799991

[17] Goto K, Morishita T, Kamada S, Saita K, Fukuda H, Shiota E, *et al.* Feasibility of rehabilitation using the single-joint hybrid assistive limb to facilitate early recovery following total knee arthroplasty: a pilot study. Assist Technol. doi:10.1080/10400435.2016.1219883. 27689789

